# Emotional intelligence, spiritual intelligence, depression and anxiety, and satisfaction with life among emerging adults in Israel and India: the impact of gender and individualism/collectivism

**DOI:** 10.1186/s40359-024-01806-6

**Published:** 2024-06-06

**Authors:** Ofra Walter, Jonathan Kasler, Surekha Routray

**Affiliations:** 1 Tel Hai, Kiryrat Shemona, Upper Gallile Israel; 2 University Khorda Bhubaneswar, Orissa, Khurda 751003 India

**Keywords:** Emotional intelligence, Spiritual intelligence, Depression and anxiety, Satisfaction with life, Emerging adults

## Abstract

Much research has focused on how emotional and spiritual intelligences promote well-being and help combat mental health issues. This comparative study, which was conducted in Israel and India with emerging adults enrolled in higher education, explored the relationship of emotional intelligence, spiritual intelligence, anxiety and depression, and satisfaction with life. The results in Israel showed a positive correlation of emotional intelligence with satisfaction with life, but in India, only spiritual intelligence correlated positively with satisfaction with life. In both groups, female participants scored higher on all variables than male participants. We offer initial explanations for these results.

## Introduction

There is a paucity of research on the role of gender among emerging adults in non-Western society [55]; the present study addressed this lacuna based on a comparison between India and Israel. In addition to examining two countries, it is also distinctive in its exploration of emerging adult populations in Western and Eastern cultures. The study investigated the between individualistic and collectivist communities, incorporating scholarly sources to substantiate its analyses (for example, [33, 75]).

Arnett et al. [5] discussed the uniqueness of emerging adulthood and how characteristics of this life stage pose challenges for mental health. For example, issues of identity that raise existential questions, which might continue until age 30, can be stressful. Emerging adulthood is considered a crossroads [53], but for many emerging adults, anxiety and depression negatively affect their efforts to reach adulthood [45]. Therefore, it is vital to assess what helps emerging adults adopt a hopeful perspective regarding their lives that will also help to reduce their levels of anxiety and depression.

According to the National Institute of Mental Health [8], 10% of children and adolescents experience a severe mental disorder that is likely to cause impairment. These problems range from substance abuse and mood disorders to symptoms of depression that can lead to suicide attempts and death. A variety of studies on college students (e.g., [11, 46]) and other adult populations (e.g., [9, 10, 38]) provide evidence to support the view that religiosity and spirituality could be protective factors for young people.

Furthermore, in the National Longitudinal Study on Adolescent Health, researchers found that prayer and religion led to improved self-esteem and decreased levels of alcohol and cigarette use among adolescents [64]. In addition, Regnerus et al. [63] noted the positive effects of religiosity on adolescents’ mental health (also see [65]). Historically, religiosity and spirituality were often agreed-upon constructs within a homogeneous and closely connected society. However, in today’s cultural, ethnic, racial, and religious diversity, a widening split has developed among aspects of transcendent beliefs, religion, and spirituality [78].

Diener et al. [15] defined satisfaction with life as a general measure of subjective well-being. This measure is intended to reflect an overall appraisal of how people feel about their lives [59]. There is some evidence that a key to successful navigation of the “in-between” years [4] may be factors that impact personal and social relations, such as emotional and spiritual intelligence [7, 57]. This was also implied in Howard Gardner’s [27] article on multiple intelligences (2004) and Rachael Kessler’s [43] book on the increasing importance of spirituality in adolescence. For emerging adolescents coping with stress during this stage of their lives, fostering emotional intelligence [7] and spiritual intelligence may promote character strengths and life skills and, as a result, positively influence outcomes [58]. This study was designed to determine whether there is a functional relationship between spiritual and emotional intelligences and life satisfaction, to suggest relevant areas of intervention and prevention of anxiety and depression in emerging adolescents in two different social cultures, and to understand the interplay of gender and cultural background in these relationships.

In light of the present comparison between two groups of emerging adults, we briefly address the subject of cultural differences. Hofstede [33] pioneered the development of a set of dimensions of by which to classify national cultures, which include gender and individualism/collectivism. In their examination of the impact of gender in different societies, found a strong effect of culture on gender behaviors. Oyserman and Lee [56] reviewed evidence of the differences between collectivist (eastern) and individualist (western) societies. They found considerable support for the view that collectivist societies foster different priorities for individuals compared with individualistic societies. More recent research has indicated differences between emerging adults living in a collectivist societies and those in individualist societies (see, e.g., [6, 30]).

Recent research by Matud and her associates shed light on differences by gender in well-being among emerging adults [49] and the role of gender in stress [50]. However, there is a paucity of research on gender among emerging adults focusing on non-western societies [55]. Therefore, in the present study we focused on gender and the collectivist/individualist dichotomy in relation to country of origin, to gain further understanding of how these cultural factors impact each of the research variables and affect their interrelationship.

### Emotional intelligence and satisfaction with life

Emotional intelligence (EI) is conceptualized as a constellation of emotional perceptions (i.e., trait emotional intelligence) or a set of skills to process emotionally related information [52],Mayer & Salovey). It represents the ability of individuals to make a connection between emotions and reasoning in a way that enables them to use emotions to guide their actions and use reasoning to regulate their emotions [52]. People with higher emotional intelligence cope better with the stressors and hassles of everyday living [79] and show greater resilience to changes under stress [70]. Emotionally intelligent individuals can cope with multiple work demands, readily shift priorities, adapt their responses and tactics to fit fluid circumstances, and respond to emotional stimuli from the inner self and the immediate environment [71]. People with a high level of emotional intelligence can achieve a balance between pleasant distractions and aversive events as well as handling changes in their moods. Therefore, they can better monitor, reflect, and control their emotions, which may contribute to their well-being [67].

Several schools of thought have emerged regarding the definition and measurement of emotional intelligence. Although beyond the remit of this research, the efforts to streamline measurement instruments are notable (see, e.g., [35]). It is likely that in the future, such measures will supersede the well-validated ones currently in general use.

Emotionally intelligent individuals can code and decode their own and others' emotions at the time they are displayed in society. Emotional intelligence is often considered crucial to successful management. Research has shown that the emotional intelligence of leaders has a positive effect on the job satisfaction of their followers [77], and also affects a wide range of other job-related behaviors and outcomes [1, 31, 32].

Huynh et al. [36] asserted that emotional intelligence cannot be understood without cognizance of cultural context. Research has found cultural differences in the perception, expression, and regulation of emotions [17, 44, 54]. Human societies are typically either individualist, endorsing values such as emotional expression and the right to free choice, or collectivist, placing less emphasis on emotional expression as essential to well-being [73].

Human resource managers of international organizations face the challenge of developing management teams that are emotionally intelligent in diverse cultural settings. However, there has been limited research to date about cross-cultural differences in emotional intelligence. How does national culture influence the emotional intelligence of individuals? Emotional intelligence is a topic of international interest [1], but the lack of knowledge regarding the cultural impact on emotional intelligence remains to be addressed [62, 68].

Some studies have found differences between individualist and collectivist societies in the relationship of emotional intelligence and well-being [24–26, 47, 48]. A comparative study revealed less subjective well-being and lower emotional intelligence among Indian students compared with their German counterparts, where the former were considered collectivist and the latter, individualist [44]. In the present study, we assumed that there would be differences between Israel, which is an individualist society, and India, which is a collectivist society. Different cultures are likely to value emotional expression differently; therefore, it is reasonable to expect that their members will have different levels of emotional intelligence, with implications for life.

### Spiritual intelligence and satisfaction with life

According to Amram and Dryer [3], emotional intelligence refers not only to abilities that draw on emotional resources but also includes those based on spiritual resources. Spirituality is a form of intelligence that predicts functioning and adaptation, as demonstrated by correlations of spirituality with improved health or well-being [18, 19]. Spiritual intelligence is the ability to create meaning based on a deep understanding of existential questions and awareness and to use multiple levels of consciousness in problem-solving [74]. Wolman (83 p. 38.) described it as "the human capacity to address the ultimate questions about the meaning of life, and to simultaneously experience the seamless connection between each of us and the world in which we live." In addition, according to Srivastava and Misra [72], spiritual intelligence assists people in any context (corporate, community, or family) develop their spiritual awareness, capacity, and intelligence, use that intelligence to be more effective as individuals, and expand their ability to make a significant contribution to the endeavors of others. It has also been shown to incorporate all the dimensions of human life that lead to meaningful lives [20].

Alex and Ajawani [2] defined spiritual intelligence as the ability to have a meaning, purpose, and value in our lives. In addition, according to Hosseini et al. [34], "spirituality can be viewed as a form of intelligence because it predicts functioning and adaptation and offers capabilities that enable people to solve problems and attain goals" (p.439). These capabilities might then affect satisfaction with life.

Satisfaction with life is a widely used construct that directly relates to a positive feeling about life [39]. Research with emerging adults research indicated that self-regulatory processes mediated the relationship between career calling and perceived employability and life satisfaction [61]. Research that investigated cultural differences between samples of students from the East and the West revealed no significant differences in satisfaction of life scores [40]. Also, mono-cultural data studies have indicated no clear directional relationship between religion and life satisfaction [16].

The results of a study that examined gender effect suggested that the relationship between religion and life satisfaction might depend mainly on gender. All but one of the male samples produced a significant positive relationship, but religiosity was not significantly associated with life satisfaction in any female sample [69].

Based on the literature reviewed, the present research was designed as a comparative study of emerging adults who were studying in higher education in India and Israel, respectively. The study focused on determinants of well-being, as measured by the Satisfaction With Life scale and by a brief measure of anxiety and depression. The first objective of the study was to observe and better understand the impact of two well-researched measures of personal agency, emotional intelligence and spiritual intelligence, on the measures of well-being. The second objective was to focus on salient cultural factors, gender and nationality, to ascertain how they impact of the interactions between the research variables.

## Methods

### Research hypotheses

Based on the previous literature, in the present study we examined the following hypotheses.

1. Emotional intelligence, spiritual intelligence, and satisfaction with life will correlate positively with each other but negatively with anxiety and depression.
2. Gender differences will be measured in the research variables: emotional intelligence, spiritual intelligence, anxiety and depression, and satisfaction with life.
3. Gender and country of origin (India or Israel) will interact with respect to all variables.
4. Spiritual intelligence and emotional intelligence will predict higher satisfaction with life in emerging adults in India, compared with their counterparts in Israel, when controlling for gender differences.

To investigate how emotional intelligence and spiritual intelligence impact satisfaction with life, we conducted a survey in Israel and India from January to May 2022.

### Participants

The total sample consisted of 554 undergraduate students, aged 17 to 30 (*M* = 23, *SD* = 2.5); 340 (61%) were from Israel and 337 (61%) were women. In the Israeli group, 225 were women; of the 214 Indian students, 95 (44%) were women.

### Instruments

1. A demographic survey was conducted to collect data on age, gender, and nationality.
2. To measure emotional intelligence, we employed the Emotional Self-Sufficiency Scale (ESES; [44]), a 32-item self-report measure based on [51] four-branch model of emotional intelligence. The scale is composed of four 8-item subscales: perception of emotions, using emotions, understanding emotions, management of emotions. Respondents are asked to rank items on a Likert scale ranging from 1 (does not describe me well) to 5 (describes me very well). The authors reported a Cronbach’s alpha of 0.92 for reliability in their original sample,in the present research the Cronbach reliability score was 0.91.
3. To measure spiritual intelligence, we used the 24-item Spiritual Self-Report Inventory (SISRI-24; [12]. This instrument was designed to measure spiritual intelligence and consists of four domains of spirituality: critical existential thinking (CET), personal meaning making (PMP), transcendental awareness (TA), and conscious state expansion (CSE). Respondents rate the items on a 5-point Likert scale. The authors reported a Cronbach’s alpha of 0.92 for the total questionnaire; in the present research the Cronbach’s alpha was 0.85.
4. To measure depression and anxiety, we used the 4-item Patient Health Questionnaire (PHQ-4; [46]). In this questionnaire, there are two items each for anxiety and depression, rated on a Likert scale ranging from 1 (not at all) to 4 (every day). The authors reported a Cronbach’s alpha of 0.81 for the total scale and 0.80 for each subscale. In the present research, we measured a Cronbach alpha of 0.70 for the total scale.
5. We also administered the Satisfaction with Life Scale [15]. This scale has 5 items, which are ranked on a 7-point scale. The authors reported a Cronbach’s alpha of 0.82,in the present research, we measured a Cronbach’s alpha of 0.77.

### Procedure

The local IRB in Israel approved the administration of the research materials (7/2020–5, 12 July 2020). This institution in India approved the research based the decision of the local IRB in Israel. Before completing an online questionnaire, participants formally consented to participate in an anonymous study and were provided with adequate information regarding the survey, as stipulated by the local IRB.

### Statistical analysis

Internal consistency of each scale was calculated using Cronbach’s alpha. Pearson correlation coefficients were calculated to assess the relationships between all subscales. A two-sample T-test was performed to examine the difference in measures across gender and country. A two-way 2 (gender: male, female) * 2 (country: Israel, India) analysis of variance (ANOVA) was run to examine the effect of gender and country on the measures.

A series of three-way analyses of covariance (ANCOVA) was run in order to examine whether gender and country moderated the relationship between each of the explanatory variables (emotional intelligence, spiritual intelligence, and depression and anxiety) and life satisfaction. SAS for Windows 9.4 was used for all analyses. *p* < 0.05 was considered as significant.

## Results

### Relationships among the measures

The correlations between the four subscales of spiritual intelligence ranged from 0.31 to 0.58, suggesting that the score on one subscale did not correspond with the score on any other subscale and each subscale represented to a different trait of the subject. Accordingly, we decided to use the four subscales and not the total score as predictors. The correlations for the four subscales of emotional intelligence ranged from 0.49 to 0.72, supporting the assumption of consistent results in each of the scales per subject. Thus, for emotional intelligence, we decided to use the total score as a predictor. Finally, a correlation of 0.50 was found between depression and anxiety; accordingly, we decided to combine these two scales to create one scale for anxiety and depression.

Table 1 presents the correlations found between the scales examined. Anxiety and depression were positively correlated with CET and negatively correlated with PMP and emotional intelligence. Emotional intelligence correlated positively with all the subscales of spiritual intelligence. Satisfaction from life was positively correlated with emotional intelligence, as well as transcendental awareness (TA), conscious state expansion (CSE), and PMP, but not with CET. In addition, there was a negative correlation between CET and anxiety and depression.

**Table 1:** Scales, descriptive statistics, and pearson correlation coefficients, *N* = 554

Scale	M	St	1	2	3	4	5	6	7
1.CET	3.60	0.69	(0.75)*						
2.TA	3.77	0.59	0.47	(0.68)*					
3.CSE	3.49	0.69	0.37	0.46	(0.71)*				
4.PMP	3.67	0.66	0.31	0.52	0.58	(0.66)*			
5.Total anxiety and depression	2.14	0.72	0.14	-0.07^1^	-0.07^1^	-0.17	(0.70)*		
6.Emotional intelligence	3.55	0.53	0.26	0.42	0.44	0.47	-0.19	(0.91)*	
7.Satisfaction with life	4.50	1.08	0.04^1^	0.27	0.25	0.40	-0.24	0.31	(0.77)*

^1^The result is not significant at *p* < .05. All the other correlations are significant at *p* < .001

^*^Note. Cronbach’s alphas appear in parentheses

### Effect of gender and country on measured scales

In both Israel and India, the scores of the women on all measures were higher than those of the men. In Israel, there was a significant difference by gender in TA, PMP, spirituality total, and satisfaction with life. In India, a significant difference between genders was found only in PMP.

A two-way ANOVA of the effect of gender and country on the measures confirmed these findings, with no significant interaction effect of gender*country on any outcome measure. In addition, the Israeli participants scored higher in TA (*p* < 0.01), and lower in CSE and anxiety and depression (*p* < 0.05) than the Indian participants did (see Tables 2 and 3).

**Table 2:** Gender and country differences

Measures	Israel					India				
	Male *N* = 98		Female *N* = 242		*P* value	Male *N* = 119		Female *N* = 95		*p* value
	Mean	SD	Mean	SD		Mean	SD	Mean	SD	
CET	3.60	0.79	3.65	0.75	0.604	3.51	0.54	3.57	0.60	0.456
TA	3.74	0.66	3.98	0.56	0.002	3.50	0.53	3.63	0.49	0.056
CSE	3.35	0.65	3.50	0.73	0.062	3.54	0.62	3.57	0.72	0.733
PMP	3.52	0.66	3.76	0.64	0.003	3.56	0.66	3.75	0.64	0.029
Spiritual total	3.50	0.48	3.64	0.50	0.019	3.53	0.45	3.63	0.49	0.119
Anxiety/depression	2.00	0.70	2.11	0.75	0.215	2.24	0.73	2.22	0.66	0.809
Emotional intelligence	3.52	0.46	3.61	0.47	0.106	3.42	0.61	3.58	0.59	0.059
Life satisfaction	4.28	1.04	4.70	1.05	0.001	4.27	1.20	4.54	0.96	0.066

**Table 3:** Effect of gender and country on outcome measures: two-way anova, regression coefficient estimate

Outcome measures	CET		TA		CSE		PMP		Spiritual total		Anxiety/depression		Emotional intelligence		Life satisfaction	
Predictors	B	SE	B	SE	B	SE	B	SE	B	SE	B	SE	B	SE	B	SE
(Intercept)	3.51^***^	0.06	3.50^***^	0.05	3.54^***^	0.06	3.56^***^	0.06	3.53^***^	0.04	2.24^***^	0.07	3.42^***^	0.05	4.27^***^	0.10
Country [Israel]	0.09	0.09	0.25^**^	0.08	-0.20^*^	0.09	-0.04	0.09	-0.02	0.07	-0.24^*^	0.10	0.10	0.07	0.01	0.15
Gender [Female]	0.06	0.10	0.13	0.08	0.03	0.10	0.20*	0.09	0.10	0.07	-0.02	0.10	0.16*	0.07	0.27	0.15
Country [Israel]* Gender [Female]	-0.01	0.13	0.10	0.10	0.12	0.13	0.04	0.12	0.04	0.09	0.13	0.13	-0.07	0.10	0.15	0.20
Observations	554		554		554		554		554		549		554		549	
R^2^ / R^2^ adjusted	0.007 / 0.001		0.108 / 0.103		0.011 / 0.006		0.026 / 0.021		0.015 / 0.010		0.014 / 0.008		0.020 / 0.015		0.032 / 0.027	

^*^p < 0.05 **p < 0.01 ***p < 0.001

Tables 2 and 3 presents the effect results of x * country (whether the relationship of the given measure with life satisfaction was moderated by country), x * gender (whether the relationship of the measure with life satisfaction was moderated by gender), and x * country [Israel]) * gender [F] (whether the relationship of the measure with life satisfaction was moderated by gender and country). The findings indicate that country moderated the relationship between emotional intelligence and life satisfaction (*B* = 0.69, *p* < 0.01).

A series of 3-way linear regressions models was employed to test the effect of gender, country, and each of the measures X on life satisfaction. "Measures X" refers to the scales CET, AT, CSE, PMP, Spiritual Total, Anxiety Depression, and EI. Table 4 presents the results of the 3-way regressions. The most interesting findings of these regressions are the effect results X * Country, that is, whether the relationship of the measure with life satisfaction was moderated by country; X * Gender, that is, whether the relationship of the measure and life satisfaction was moderated by gender; and X * Country [Israel]) * Gender [F], that is, whether the relation of the measure and life satisfaction was moderated by gender and country.

**Table 4:** Moderation of the relationships of measures with life satisfaction by gender and country: three-way ANOVA, regression coefficient estimate (B,) and standard error (in parentheses)

Dependent variable	Life Satisfaction	Life Satisfaction	Life Satisfaction	Life Satisfaction	Life Satisfaction	Life Satisfaction	Life Satisfaction
X Predictors	CET	TA	CSE	PMP	Spiritual Total	Anxiety Depression	EI
	*B)SE(*	*B)SE(*	*B)SE(*	*B)SE(*	*B)SE(*	*B)SE(*	*B)SE(*
(Intercept)	4.36^***^ (0.64)	3.31^***^ (0.65)	1.97^***^ (0.55)	1.99^***^ (0.50)	2.04^**^ (0.75)	4.48^***^ (0.31)	3.56^***^ (0.52)
X	-0.03 (0.18)	0.27 (0.18)	0.65 ^***^ (0.15)	0.64^***^ (0.14)	0.63^**^ (0.21)	-0.10 (0.13)	0.21 (0.15)
Country [Israel]	-0.04 (0.81)	-0.92 (0.89)	0.87 (0.78)	-0.48 (0.73)	0.01 (1.07)	0.48 (0.44)	-2.46^**^ (0.94)
Gender [F]	-2.46^**^ (0.94)	-0.83 (1.05)	1.86^*^ (0.78)	1.04 (0.79)	-0.56 (1.11)	0.19 (0.49)	-0.14 (0.85)
X * country [Israel]	0.01 (0.23)	0.23 (0.24)	-0.22 (0.22)	0.15 (0.20)	0.00 (0.30)	-0.24 (0.20)	0.69^**^ (0.27)
X * gender [F]	0.76^**^ (0.26)	0.29 (0.29)	-0.45^*^ (0.21)	-0.24 (0.21)	0.21 (0.31)	0.04 (0.21)	0.10 (0.24)
Country [Israel] * Gender [F]	3.09^**^ (1.11)	1.33 (1.31)	-1.17 (1.01)	-0.33 (1.03)	1.50 (1.44)	0.77 (0.61)	0.06 (1.27)
X * country [Israel])* gender [F]	-0.82^**^ (0.31)	-0.34 (0.35)	0.35 (0.28)	0.11 (0.28)	-0.38 (0.40)	-0.28 (0.27)	0.01 (0.35)
Observations	549	549	549	549	549	549	549
R^2^ / R^2^ adjusted	0.060 / 0.048	0.088 / 0.076	0.099 / 0.087	0.182 / 0.171	0.105 / 0.094	0.113 / 0.102	0.150 / 0.139

^*^p < 0.05 **p < 0.01 ***p < 0.001

*Note*. X: CET, TA, CSE, PMP, Spiritual Total, Anxiety Depression, EI

**Fig. 1:**
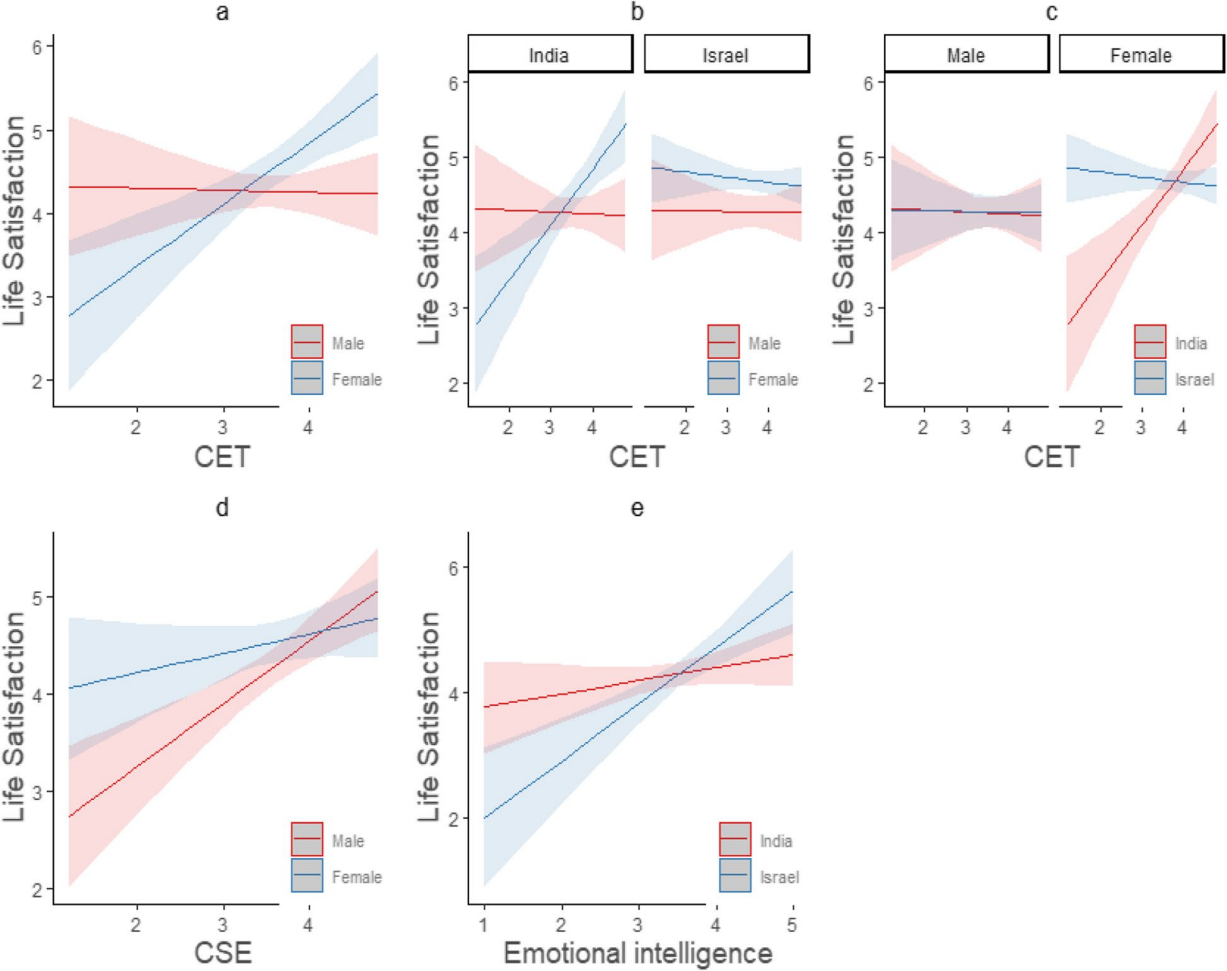
Significant Interaction Results of 3-Way ANOVA

According to these findings, country moderated the relationship between emotional intelligence and life satisfaction (*B* = 0.69, *p* < 0.01). Figure 1 provides a visual representation of the relationships found between variables of the present research. Panel e shows that the relationship between EI and life satisfaction was stronger in Israel than in India. Panel b shows that that gender moderated the relationship of CET and life satisfaction (B = 0.76, *p* < 0.01),

The relationship of EI and life satisfaction was stronger in Israel than in India (Fig. 1, Panel e). Gender moderated the relationship of CET and life satisfaction (B = 0.76, *p* < 0.01); among the female respondents, there was a positive correlation between CET and life satisfaction, but among the male respondents, there was no significant correlation (Fig. 1, Panel a). Gender also moderated the relationship of CSE with life satisfaction (B = -0.45, *p* < 0.05); it was stronger among the male respondents compared with the female respondents (Fig. 1, Panel d).

Country and gender both moderated the relationship between CET and life satisfaction (B = -0.82, *p* < 0.01). The only positive correlations between these measures were found among the female respondents in India (Fig. 1, Panels b and c). More specifically, the correlation between CET and life satisfaction was high among women in India, but there was no such correlation among respondents of either gender in Israel (Fig. 1, Panel b), nor among men in either country (Fig. 1, Panel c).

## Discussion

In this research we compared emerging adults who were enrolled in institutions of higher education in in India and Israel. The primary research question concerned the relationship of emotional intelligence, spiritual intelligence, and anxiety and depression with satisfaction with life.

Our first hypothesis was that emotional intelligence, spiritual intelligence, and satisfaction with life would correlate negatively with anxiety and depression and that there would be positive correlations between all other variables. However, the results revealed a complex relationship between spiritual intelligence and anxiety and depression.

Anxiety and depression correlated positively with critical existential thinking and negatively with personal meaning production, two domains of spiritual intelligence. Furthermore, no statistically significant correlations were found with the other scales of spiritual intelligence. Interestingly, Giannone and Kaplin, [29] arrived at similar results. Furthermore, in previous research [42] we also found that the critical existential thinking of emerging adults correlated positively with depression and anxiety. A possible explanation for this might be that a critical examination of existential issues can lead to elevated levels of anxiety and depression. Accordingly, these results might be explained by the low correlations between the scales of spiritual intelligence. Thus, the construct of spiritual intelligence should be viewed with caution. At present. it is prudent to conclude that subscales of spiritual intelligence seem to form a loose set of related measures, rather than being aspects of a unified construct of spiritual intelligence.

In contrast, as in our previous research [42], emotional intelligence correlated positively with satisfaction with life and negatively with depression and anxiety. In this case, these results fall in line with relatively high correlations between subscales of emotional intelligence measured in the present research and reported in the literature [37].

To delve deeper into the relationships between variables, we focused on the cultural issues of gender and country of origin to determine how these demographic elements affected the results. Therefore, our next two hypotheses (2–3) represented expectations of variations between genders as well as differences between emerging adults in India compared to those in Israel. The findings indicate that the female participants scored higher than their male counterparts on all measures, irrespective of country of origin.

There is some controversy regarding gender differences in emotional intelligence. Petrides and Furnham [60] found that men estimated that they had higher emotional intelligence than women did. Fernandez-Berrocal [23] also found evidence that men reported higher emotional intelligence than women did, but according to their results, women scored higher than men on ability measures. In later work, [22] found that age interacted with gender when measuring emotional intelligence. Finally, more recently, Salavera et al. [66] did not find gender differences in emotional intelligence in their research. Our results, based on a self-report measure of emotional intelligence, add to the discussion, but there is still no clear-cut conclusion on this subject.

With regard to gender differences in spiritual intelligence, there is also no consensus in the research on this matter. For example, Pant and Srivastava [58] found little gender variation in spiritual intelligence, but there is sparse literature on this subject. We note that in spite of a complex relationship between subscales of spiritual intelligence, gender differences remained consistent in each of the subscales of spiritual intelligence.

With regard to satisfaction with life, the literature is also inconclusive (for example, both Joshanloo [41] and Della Giusta et al. [13] found no consistent differences in gender. Our results do not reflect the current literature, it may be that specific populations as those in the present study present different results. We note that Della Giusta et al. [13] reported that women in employment presented higher levels of satisfaction with life than men. Moreover, these results align with those of a previous study that employed a similar measure [76]. Furthermore, de Vibe et al. [14] found that Indian females registered higher values on the subjective well-being scale (i.e., cognitive well-being) than Indian males did.

Our comparison of Indian and Israeli emerging adults (Hypothesis 4) yielded interesting results. We found that emotional intelligence predicted satisfaction with life in the Israeli sample but not in the Indian sample, but spiritual intelligence had much higher predictive power on satisfaction with life in the Indian sample than the Israeli group. This might suggest that more collectivist societies, such as that in India, are more attuned to spirituality, compared with more individualistic western societies, such as Israeli society, where emotional intelligence is more significant. Previous research found compelling evidence for this view [76].

Furthermore, spirituality is a particularly strong aspect of Indian culture, and it should come as no surprise that our results reflect this. Indian culture is a collectivist society that emphasizes group cohesion values and spiritual practice is part of their culture. In contrast, Israeli society is individualistic and values the freedom to pursue personal fulfillment; this, in turn, may have a positive impact on mental well-being [75]. Germani et al. [28] found a significant effect of country on satisfaction with life,where the higher the country's individualized score, the higher the average satisfaction with life score, in the following order: Americans, Italians, Russians, and Chinese. At the individual level, satisfaction with life was unrelated to individualism, which consists of desiring to be unique, distinct from groups, and being highly self-reliant. Instead, it was associated with collectivism, which concerns the wish to see oneself as being similar to others and emphasizes common goals with others, interdependence, and sociability, without the need for submission to authority. The positive link between satisfaction with life and collectivism suggests an essential role of family connectedness in satisfaction with life across different cultures during emerging adulthood. This might explain the effect of critical existential thinking, one of the components of spiritual intelligence, on Indian emerging adults' satisfaction with life, which is one of the values of the Indian collectivist society In contrast, among Israeli emerging adults living in a more individualistic country that emphasizes more the self and emotional abilities, we found emotional intelligence to be a strong predictor of satisfaction with life.

## Conclusion

This study compared emerging adults in India and Israel in terms of the relationship between emotional and spiritual intelligence and satisfaction with life and anxiety and depression.

The results indicated differences between the two groups of participants in terms of the role of spirituality and emotional intelligence in predicting satisfaction with life and anxiety and depression. We suggest that these the differences are related to the collectivist/individualist orientation of the two societies. Traditional eastern values encourage emotional restraint in deference to the needs of the community, while western values encourage expressing the individual needs of self, including emotional expression. Among the Israeli emerging adults, emotional intelligence correlated positively with satisfaction with life but negatively with depression and anxiety. In contrast, critical existential thinking, a component of spiritual intelligence, correlated positively with satisfaction with life, but only in Indian females.

This comparative research provides some insights in two cultural issues: the impact of gender difference and that of differences in country of origin. A deeper look at the concept of cultural differences as expressed by individualist-collectivist societal orientations could clarify and perhaps better explain results of surveys comparing different societies. The results of recent studies indicate the need for a more nuanced investigation when using this concept as a classification of societies (see, e.g., [21], and [28]).

Future research should also examine emotional intelligence and spirituality intelligence as traits or abilities, to promote better understanding of how they are related to satisfaction in life as well as depression and anxiety among emerging adults in different cultures. Our results also provide evidence that may inform the search for means to decrease depression and anxiety.

## Recommendations

Research in the field of identifying skills and abilities that contribute to well-being among emerging adults has indicated the possible role of interventions that promote emotional intelligence and aspects of spiritual intelligence, particularly production of meaning in life. Such interventions might help ameliorate tendencies towards anxiety and depression and provide pathways to greater satisfaction with life. Such interventions should be sensitive to cultural aspects of society in order to attain goals of enhancing well-being among emerging adults. The study of such efforts would be of value to researchers and practitioners around the globe and help in tailoring interventions to varying cultural settings. Thus, the present study is relevant to international audiences working in a variety of settings.

## Data Availability

Not applicable.
